# Overcoming Obstacles: Perspective on How Mediterranean Oaks Defend Their Acorns from Insect Seed Predators

**DOI:** 10.3390/insects16090990

**Published:** 2025-09-22

**Authors:** David A. Oropesa-Olmedo, Enrique Andivia, Michał Reut, Pablo Cisneros, Raúl Bonal

**Affiliations:** 1 Department of Biodiversity, Ecology and Evolution, Faculty of Biological Sciences, Complutense University of Madrid, 28040 Madrid, Spain; eandivia@ucm.es (E.A.); pablocis@ucm.es (P.C.); rbonal@ucm.es (R.B.); 2 Section of Applied Entomology, Department of Plant Protection, Institute of Horticultural Sciences, Warsaw University of Life Sciences-SGGW, Nowoursynowska 159, 02-776 Warsaw, Poland; michal_reut@sggw.edu.pl

**Keywords:** acorn, weevils, phenology, *Quercus*, tannins, pre-dispersive predation, infestation

## Abstract

Insect pre-dispersal seed predators attack a large proportion of the acorn crops in oak forests worldwide. Oaks (*Quercus* spp.) have evolved several strategies, including physical barriers, chemical defenses (e.g., tannins), and/or phenological predator avoidance, to reduce infestation rates. This study examines how four Mediterranean oak species cope with acorn-feeding insects. Nearly 4000 acorns were collected from five sites at two time points during the maturation period: in mid-September and mid-October. Infestation rates were higher in mid-September, when the pericarp is softer and easier to drill, but at that time, the cotyledon tannin content was higher. *Q. coccifera* acorns had the highest tannin concentration, which, we experimentally discovered, hampered weevil development (with longer development and a lower final larval mass). Infested acorn abscission was also more effective in *Q. coccifera*. Due to the smaller size and later maturation phenology of its acorns, insects depleted the cotyledons and suffered food shortages more frequently. In the end, *Q. coccifera* showed the lowest acorn infestation rates, although its strategy would have costs in further stages of the regeneration cycle. Tannins deter acorn dispersers, and their production is costly. Such trade-offs would favor the co-existence of different strategies evolved by *Quercus* spp. against pre-dispersal insect predators.

## Simple Summary

Oak seeds (acorns) are an important food source for insect larvae, such as acorn weevils, moth caterpillars, and gall wasps, but they have evolved several strategies to defend themselves from these seed predators. This study looks at how four types of oak trees, *Q. coccifera*, *Q. ilex*, *Q. faginea*, and *Q. pyrenaica*, use different defenses to protect their acorns. These defenses include chemical compounds like tannins, physical traits, and the timing of acorn development. We collected nearly 4000 acorns from five different locations at two different stages of growth and analyzed the rate of infestation, the tannin content, and the size of the acorns. We also examined how larvae of a specific acorn weevil, *Curculio elephas*, developed in the acorns of each oak species. The results showed that oak species with higher tannin levels, like *Q. coccifera*, had fewer pests and slower larval development. These findings help us understand how plants and insects interact and how oak trees balance protecting their acorns with their need to spread seeds, which can be important for maintaining healthy ecosystems.

## 1. Introduction

Pre-dispersive seed predation results in a significant loss of reproductive potential in plants [1,2,3,4]. Seeds are a very nutritious food resource, as they contain the reserve substances to be used by the seedlings during their early life stages [5]. For this reason, plants have evolved strategies to avoid or reduce the impact of pre-dispersive seed predation, which in turn have sometimes triggered co-evolutionary arms races between plants and animals [3,6,7].

Seed defenses include both mechanical barriers and biochemical compounds. The first ones refer to physical barriers (like hard pericarps and/or those with a distinctive shape) and the latter to secondary metabolites, which are toxic or deterrent for predators [8,9,10]. Biochemical defenses include substances like tannins, secondary metabolites that can reduce the digestibility of proteins, thereby decreasing the nutritional value of plant material and hampering insect growth and development. Besides defenses, plants have evolved tolerance strategies involving predator satiation [11]. Large crops can satiate predators, especially when seed production is synchronized among conspecifics and variable among years (a phenomenon referred to as masting) [12,13]. In addition, satiation may occur at the seed level, when the cotyledons are large enough to satiate predators before reaching the embryo, thus allowing germination and further seedling development [14,15,16].

Saving resources from being invested in seeds with lower recruiting chances underlies another strategy against granivore insects, namely, premature seed abscission [17,18]. Plants are able to distinguish herbivory from mechanical damage [19] and can also detect insect oviposition. In the case of seed predator larvae, such as *Curculio elephas*, suppression of seed growth due to abscission reduces the final larval size by limiting the amount of available cotyledon, which negatively affects insect fitness [18]. Other strategies do not require changes in resource allocation but consist of a sort of phenological “hide and seek” game. In many bioclimatic regions, seeds are only available and vulnerable to insect attack during a brief period of time [20]. In turn, insect biological cycles have evolved to overlap with that timing as much as possible. However, plants may sometimes mature and release seeds during periods in which other factors (e.g., climatic) do not allow the presence of insects, thus avoiding oviposition or seed predation to some extent [21,22,23].

**Figure 1:**
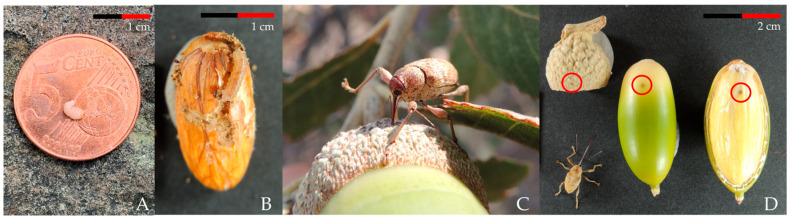
(**A**) The appearance of a *C. elephas* larva on a coin (diameter: 21.25 mm). (**B**) *Cydia* spp. larva feeding on an acorn cotyledon, with feeding marks inside. (**C**) *C. elephas* female drilling a hole for oviposition. (**D**) Acorns with oviposition holes (circled in red) made by a *C. elephas* female; the large rostrum used for boring is clearly visible.

In this study, we will assess the defensive strategies of different oak species (*Quercus* spp.) against the insects that predate their seeds before dispersal. In oaks, similarly to other Fagaceae like beech (*Fagus* spp.) or chestnut trees (*Castanea* spp.), the main physical defense of their seeds is the pericarp. Oak seeds (acorns) have a pericarp that protects the cotyledons and embryo against many insects and small birds. However, there are specialist pre-dispersal insects that are able to feed on them, mainly weevil larvae, *Curculio* spp. Linnaeus (Coleoptera: Curculionidae; Figure 1A), moth caterpillars, *Cydia* spp. Hübner (Lepidoptera: Tortricidae; Figure 1B), and larvae of gall-forming wasps, *Callirhytis* spp. Förster (Hymenoptera: Cynipidae). These insects attack acorns before they are fully ripe following different strategies. In the case of *Cydia* spp., the newly hatched larvae are able to enter the acorn, whereas in *Callirhytis* spp. and *Curculio* spp., adults drill the pericarp and oviposit into the cotyledons (Figure 1C) [18,24,25]. Female *Curculio* spp. have a disproportionately long rostrum, which allows them to drill a tiny puncture through the seed coat and introduce their ovipositor into the acorn (Figure 1D) [26].

The pericarp may thus not stop all insects, but acorns also have chemical compounds (e.g., tannins), which could act as defenses. Accordingly, several studies have identified significant differences in the cotyledon tannin content both among oak species and in relation to geographical and biometric parameters [27,28]. In addition, the tannin concentration seems to decrease with acorn maturation, although this effect is species-specific [29,30]. Oaks can also satiate predators at the tree level (large crop sizes) and at the seed level (large acorn cotyledons), although the same individual cannot combine both due to seed size/number trade-offs [1,31]. Moreover, Espelta et al. 2009 [14] also showed the role of oak phenology on susceptibility to *Curculio* weevil damage. Acorns become more vulnerable to insect infestation once they have achieved a certain size (threshold to allow larval growth) [32] and the pericarp has not turned too hard to be drilled [20]. However, *Curculio* spp. phenology cannot always be coincident with the best timing for attacking acorns, since other factors (e.g., rainfall timing) influence it strongly [33]. Thus, oak species in which acorn growth overlaps with insects’ optimum phenology would be expected to have higher infestation rates.

Here, we focused on pre-dispersal acorn predation by insects in four Iberian oak species with contrasting ecological requirements, namely, kermes oak (*Quercus coccifera* L.), holm oak (*Q. ilex* L.), Portuguese oak (*Q. faginea* Lam.), and Pyrenean oak (*Q. pyrenaica* Willd.). The main aim was to analyze the effectiveness of chemical, mechanical, and phenological avoidance strategies against pre-dispersal acorn predation in each species. We predict that those oak species with the worst conditions—small acorn size and high tannin content—for larval development would show lower acorn infestation rates. The specific objectives of this study were (1) to analyze the dynamics of infestation rates throughout the acorn development and maturation period for each species; (2) to evaluate interspecific differences in tannin concentrations in acorn cotyledons, as well as their variability across the development and maturation period; (3) to assess the development of *Curculio elephas* larvae in acorns of different *Quercus* spp. through a manipulative experiment; (4) to determine interspecific differences in acorn growth phenology and their implications for predator satiation and insect damage via premature acorn abscission.

## 2. Materials and Methods

Sampling was carried out at five sites in the center of the Iberian Peninsula (Table 1). The area has a Mediterranean climate, with temperate, humid winters and warm-to-high temperatures and dry summers [34]. There were, however, differences in the climatic conditions among the study sites associated with altitude, which also determine the occurrence of the study species (Table 1).

Oak species in the Iberian Peninsula show distinct ecological preferences and altitudinal distributions linked to regional climatic differences. *Q. ilex* and *Q. coccifera* are evergreen species typical of the Mediterranean basin. *Q. ilex* has a broad climate tolerance, occurring from sea level to 2000 m, in both xeric and humid climatic conditions. *Q. coccifera* is more restricted to xeric areas and occurs from sea level up to 1500 m. *Q. faginea* and *Q. pyrenaica* are deciduous species associated with moist conditions. *Q. pyrenaica* is mostly restricted to humid areas and occurs at elevations between 740 and 1300 m. *Q. faginea*, found between 200 and 1900 m, is also present in humid regions but can grow in less rainy areas, as long as water availability remains relatively stable throughout the year [36].

### 2.1. Field Sampling

At each site, we collected acorns in two periods separated by one month. The first harvest took place in mid-September 2023 (maturation period T1), and the second in mid-October 2023 (maturation period T2). Acorns are finishing their maturation at this time of the year; their growth is still ongoing until they reach their final size [18], and their color is turning from vibrant green to brown. Moreover, the decrease in tannin content (see below) confirmed the maturation progress over the study period, as tannin decrease is a general trend in ripening fruits [37,38]. We randomly selected eight trees of each species per site and randomly collected around 20 acorns per tree. A total of 192 trees were selected, and 4088 acorns were finally collected between T1 and T2. We took acorns directly from the branches to estimate infestation rates more accurately [17,18,39,40]. Collecting acorns from the tree branches allowed us to estimate infestation rates and also recent weevil oviposition activity, as infested acorns remain attached to the branches an average of 15 days before being prematurely abscised [41]. In addition to the aforementioned acorns, we collected three more per tree without infestation marks from each selected tree to analyze their tannin concentration and determine how it varied among *Quercus* spp. and between T1 and T2 [41]. We oven-dried these acorns for 48 h at 80 °C. Then, we pooled the acorns without mixing sites, species, and periods and grinded them in a ball mill. The percentage of tannins in the cotyledons was analyzed at the Servicio de Apoyo a la Investigación de Extremadura (SAIUEx) following the casein method [42].

### 2.2. Larva Monitoring

Firstly, we classified acorns as healthy or infested based on the presence of marks on the acorn pericarp. *Curculio* spp. drilling causes a dark spot on the seed coat, usually under the acorn cupule. *Cydia* spp. produces an irregular mark similar to a scratch under the cupule. Although we were unable to visually distinguish any markings on the pericarp, *Callirhytis* spp. produces wrinkles and, in some cases, cracks on the acorn’s surface [25]. In any case, to rule out any misidentification, we allowed larvae to complete their development and so confirm infestation. To do so, all the acorns were placed in individual transparent plastic bottles with smooth walls and a volume of 125 mL for two months at a constant temperature of 18 °C in the dark using a laboratory incubator. After this period, they were kept at room temperature (22 °C) for a further month. Acorns were checked weekly to visually monitor the emergence of larvae, because upon emergence, larvae drill a hole through the acorn shell. We could thus know the proportion of acorns attacked by each insect taxa (Table 2).

However, for the analyses, we used as the dependent variable the global rates of infestation by insects (instead of dividing them by taxa). We did this for several reasons. Firstly, even if they are not the majority, there are some mixed infestations (acorns infested by *Curculio* spp. and *Cydia* spp., for example), so in some cases, the acorn infestation cannot be attributed to a single taxon. Moreover, with the exception of the rare gall-making *Callirhytis* spp., both *Cydia* spp. and *Curculio* spp. larvae feed on the cotyledons in a similar way, may deplete cotyledons, etc. The response of the oaks to their attack is the same, too, namely, the premature abscission of the infested acorn [18].

After three months of monitoring, we did not expect to find live larvae, as their development is usually finished in one month [43]. To standardize the moisture content, we oven-dried all acorns (with and without an exit hole) at 80 °C for 48 h in order to take morphometric measurements. Then, we opened up the dried acorns to confirm whether those without holes were infested and checked whether insects had depleted the cotyledons and predated the embryo or not in the infested ones. In summary, acorns with larval exit holes were undoubtedly classified as infested, as well those from which no larvae had emerged but there were dead larvae, *Callirhytis* galls, or *Curculio* and *Cydia* excrement [40].

### 2.3. Acorn Volume

We selected three infested acorns from each maturation period (T1 and T2) and tree and measured their length and width before opening them up after the oven-drying procedure previously described. We measured them using a precision caliper gauge to an accuracy of 0.01 mm. We used these measurements to calculate the mean acorn volume for each tree, using the ellipsoid volume formula: V = (4π/3) × r1 × (r2)^2^ [40]. This variable is highly relevant as it is directly related to food availability (cotyledon volume). We measured three acorns per tree based on previous studies that concluded that acorn size for a specific year is highly consistent at the intra-individual level [1].

### 2.4. Development Monitoring of Acorn Weevil Larvae

To assess the performance of insects in acorns of different oak species, we conducted a manipulative experiment using *Curculio elephas* larvae as a model. We chose *Curculio* spp. for the experiment because their legless larva are more appropriate for the experimental transfer among acorns of different *Quercus* spp. In contrast, *Cydia* spp. caterpillars have legs and are more likely to leave the experimental acorns prematurely. Following the method of Reut et al., 2023 [44], we extracted 120 *C. elephas* larvae in their early stages of development (between 2 and 3 mg) and transferred then to sound acorns. We extracted all larvae from *Quercus ilex* acorns from Huecas to ensure that all individuals belonged to the same species, as *C. elephas* was the only weevil species at that study site [45]. We used 30 sound acorns per *Quercus* species to transfer the larvae. We selected acorns without oviposition punctures or any sign of insect infestation on the acorn shell. We avoided collecting any of these acorns in Huecas, to eliminate any potential confounding effect of local adaptation. We confirmed that no infested acorn was chosen in error, as no larvae but the one transferred emerged from each experimental acorn. In each sound experimental acorn, we drilled a tiny hole through the seed coat and into the cotyledons, inserted one larva, and closed the hole with plasticine. Then, we placed each experimental acorn in individual 125 mL transparent plastic bottles with one centimeter of regularly moistened sand and kept them at room temperature. We checked the acorns daily and recorded the emergence date and larval weight upon emergence. We weighed each larva with a precision balance (to the nearest 0.1 mg) both at the beginning and at the end of the experiment. This allowed us to determine the final larval weight, the length of the development period (in days), and the mass gain rate (in milligrams per day).

### 2.5. Statistical Analysis

First, we analyzed whether acorn infestation rates differed between study species and acorn maturation periods (objective 1). For this, we fitted a generalized linear mixed model (GLMM) with a binomial distribution and a logit link function and considered the pairwise interaction between species identity and maturation period as a fixed effect. Locality was considered as a random term in the model to account for the lack of independent results for sample trees within each location. To assess how the infestation rate varied among the study species, we applied post hoc pairwise multiple comparisons of the estimated marginal means using Tukey’s adjustment.

Second, we evaluated how tannin concentration in the cotyledon of acorns varied across species and maturation periods (objective 2) by fitting a Linear Model (LM) including species identity and maturation period as fixed factors. To compare species-specific variations in tannin concentration across periods, we also conducted Tukey’s post hoc test.

Third, we also evaluated whether the length of the development period and the final larval mass differed among weevil (*C. elephas*) larvae growing within acorns of different *Quercus* spp. (objective 3). For this, we used an LM for each response variable (length of the development period and final mass of the larvae) with oak species identity as a fixed effect. Then, we performed post hoc analyses to assess significant differences between *Quercus* spp. Finally, to assess interspecific differences in acorn volume related to the maturation period and their implications for predator satiation and insect damage to the acorn embryo (objective 4), we performed an LM with the pairwise interaction between species identity and maturation period as a fixed effect. The acorn volume was log-transformed to achieve homoscedasticity. We also evaluated whether the proportion of acorns in which cotyledons were depleted and the proportion in which the embryo was damaged by larvae differed among *Quercus* spp. To do so, we fitted two separate generalized linear models (GLMs) with a binomial distribution and a logit link function considering the oak species identity and maturation period as fixed factors. Differences between *Quercus* species were then analyzed using Tukey’s post hoc tests. Differences between maturation periods were assessed using a pairwise comparison of the estimated marginal means obtained from a binomial generalized linear model. As only two time points were compared, no multiple comparison adjustment was necessary. We used the R environment for all the analyses and the packages lme4 [46] and emmeans [47].

## 3. Results

### 3.1. Infestation Rates of Acorns Among Oak Species and Between Maturation Periods

**Figure 2:**
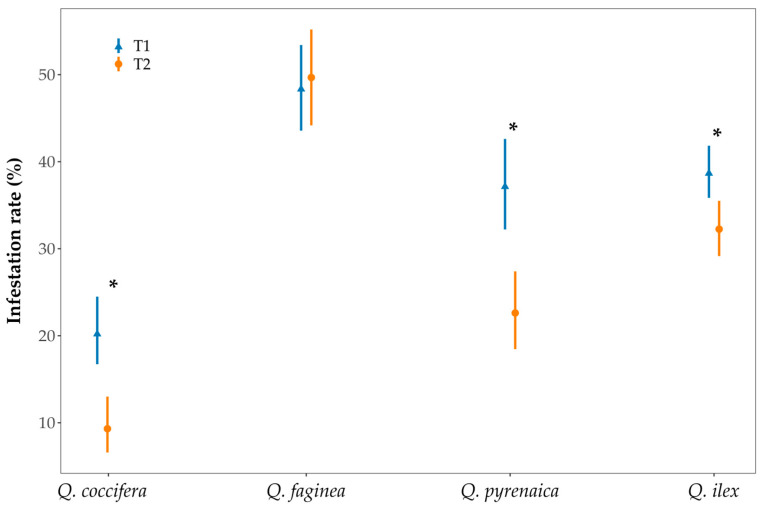
Infestation rate (%) per species and maturation period (T1–T2). Means and 95% confidence intervals (CIs) are shown for each species identity. Asterisks showed significant differences between maturation period within species.

We found a significant interaction effect between oak species and maturation period on the infestation rates of acorns (χ^2^ = 19.201, *p* < 0.001). Overall, infestation rates decreased significantly throughout the season in all species studied except for *Q. faginea* (Figure 2).

### 3.2. Differences in Tannin Concentration Between Species and Maturation Periods

**Figure 3:**
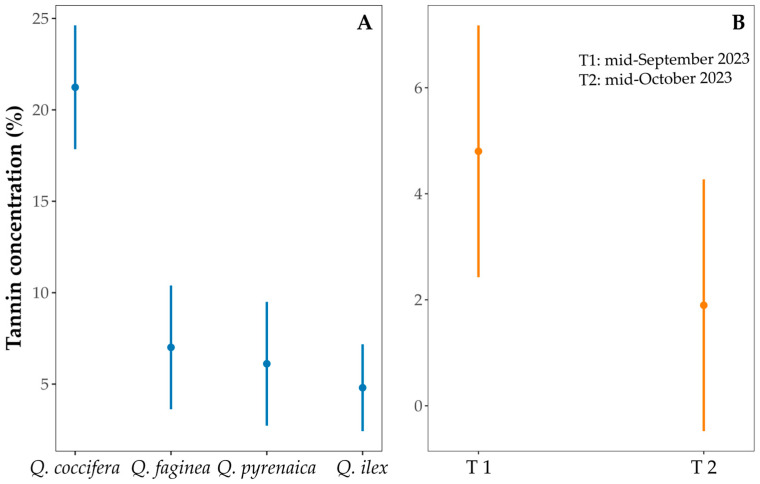
Tannin concentration (%) for each oak species (**A**). Tannin concentration (%) for each maturation period (**B**). Means and 95% CIs are shown for each species.

We observed a significant effect of oak species identity (*F* = 31.25, *p* < 0.001) and maturation period (*F* = 5.325, *p* < 0.05) on tannin concentration in the cotyledons. Tannin content was highest in *Q. coccifera* acorns compared to the rest of the species (all pairwise comparisons *p* < 0.001; Figure 3A). Tannin concentration also decreased over the maturation period, being higher in T1 than in T2 (Figure 3B). The decrease in tannin concentration occurred in all oak species and localities from T1 to T2 (Table 3).

### 3.3. Larval Development in Acorns of Different Oak Species

**Figure 4:**
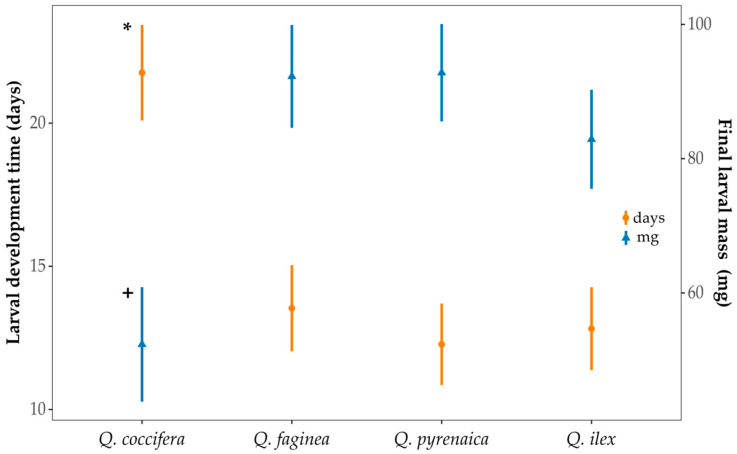
Left axis with orange circles and error bars: larval development time (days). Right axis with blue triangle and error bars: final larval mass (mg). Means and 95% CIs are shown for each species. *Q. coccifera*, marked with an asterisk and a cross, showed significant differences in larval development time and final larval mass compared to the other species.

Larval development duration differed among oak species (*F* = 30.229, *p* < 0.001) and took significantly longer in *Q. coccifera*, in which the number of days needed to complete growth was higher compared to all other species (*p* < 0.001, Figure 4). The final larval mass was significantly lower in acorns of *Q. coccifera* than in the rest of the *Quercus* spp. (*p* < 0.001): the pairwise comparisons between the other species were not significant (Figure 4). Larval survival during development for the study species was 70.0% in *Q. coccifera*, 93.3% in *Q. ilex*, 86.7% *in Q. faginea*, and 96.7% in *Q. pyrenaica*.

### 3.4. Acorn Volume and Predation Rate of Cotyledon and Embryo

The final volume of acorns was significantly influenced by maturation time (*F* = 19.02, *p* < 0.0001), species identity (*F* = 8.16, *p* < 0.0001), and their interaction (*F* = 4.74, *p* = 0.0035). In this regard, only acorns of *Q. coccifera* (*p* = 0.0049) and *Q. pyrenaica* (*p* < 0.0001) showed significant increases in volume between maturation periods (Table 4).

**Figure 5:**
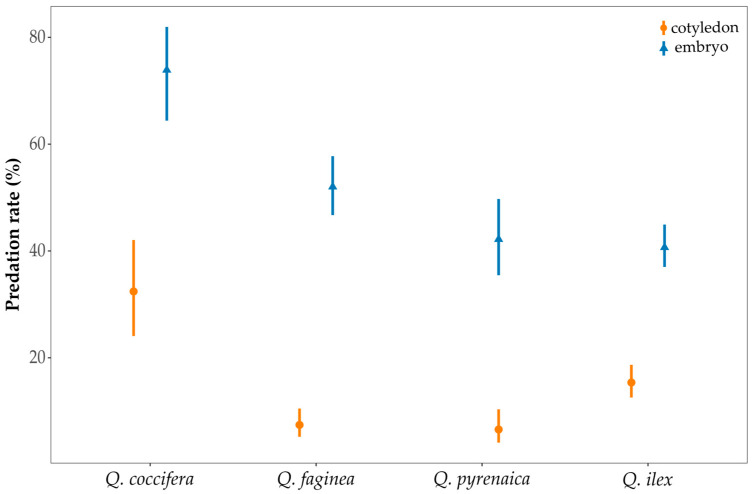
Orange circles and errors bars: cotyledon predation rate (%). Blue triangle and error bars: embryo predation rate (%). Means and 95% CIs are shown for each species.

The proportion of acorns in which the cotyledons were completely depleted by insects differed significantly among oak species (*χ*^2^ = 69.57, *p* < 0.0001) and over the maturation period (*χ*^2^ = 98.39, *p* < 0.0001). Cotyledons were completely depleted more frequently in T1 (26.4 ± 1.9%) than in T2 (5.83 ± 1.1; *p* < 0.0001). Among species, the highest percentage of acorns with no cotyledons left was registered in *Q. coccifera* (32.4 ± 4.6%), while *Q. pyrenaica* showed the lowest (6.6 ± 1.6%; Figure 5).

The embryo predation rate also differed among oak species (*χ^2^* = 48.36, *p* < 0.0001) and the maturation period (*χ^2^* = 118.46, *p* < 0.0001). The embryo predation rate was significantly higher in T1 (68.1 ± 2.0%) than in T2 (37.2 ± 2.5%; *p* < 0.0001). Among oak species, the percentage of acorns in which the embryo was predated by insects was highest in *Q. coccifera* (74.1 ± 4.5%), while *Q. ilex* showed the lowest (40.9 ± 2.0%; Figure 5).

## 4. Discussion

Tannins delay and constrain larval growth [48], suggesting that oaks maintain high levels of tannins in their organs as a defensive response to intensified herbivory pressure. As shown in seeds and fruits of other tree species [49], tannin concentrations also decreased over the acorn development period. Acorn tannin concentrations were higher at T1 (second half of September), when acorn infestation rates are higher. This early insect attack on tannin-richer acorns might seem contradictory; however, there may be different factors that select against a later oviposition. In first place, insect phenology is largely conditioned by meteorology, for example, in acorn weevils [33]. Moreover, competition for seeds (oviposition sites) would select against a delayed oviposition timing [33]. Also, late in the season, the acorn pericarp is fully sclerotized and thus more difficult for insects to drill [20]. If the phenology of acorn growth is brought forward due to climate change, seed predators will find that the pericarp has hardened and therefore the fitness of these insects will decrease due to the impossibility of oviposition [33].

The evergreen *Q. coccifera* showed the highest tannin concentration in acorns compared to the rest of the studied oak species. *C. elephas* larvae developed poorly in *Q. coccifera* acorns, which contained three times more tannins than the rest of the oak species. Moreover, the extended development period that larvae spent within *Q. coccifera* acorns could not compensate for the poor nutritional quality of its cotyledons, and the final larval mass was significantly lower compared to all other *Quercus* spp. The negative effects of tannins on insect growth have been demonstrated in other plant species [50,51]. In the case of oaks, the worst larval performance within *Q. coccifera* acorns would have further negative fitness consequences, as larval mass is strongly correlated with survival likelihood, adult size, and female fecundity [52]. In addition, a prolonged period within prematurely dropped infested acorns would increase intraguild predation on larvae by ungulates [41].

The negative effects of acorn tannins on weight gain have been reported in vertebrates [53,54], but, to the best of our knowledge, this is the first time that they have been shown in insects. The effects depend on different factors such the tannin chemical type [55] or the interaction with other compounds (e.g., proteins or lipids), which may enhance or reduce their negative consequences [53,56]. In contrast with other studies [56], we did not test the effects of tannins by adding them to the acorns. However, due to the large differences in acorn concentration between species, we hypothesized that the poor larval development in acorns of *Q. coccifera* could be related to their high tannin concentration.

Infestation rates were highest at T1, when acorns are still developing. At that time, acorns are easier to drill [20] but also smaller, meaning that seed predator larvae deplete the cotyledons more frequently and may face food restriction [18]. Insect larvae faced the strongest food limitation in *Q. coccifera* acorns, in which the cotyledons were completely depleted more frequently than in the rest of the *Quercus* spp. *Q. coccifera* acorns were not only the smallest globally but also proportionally smaller at T1 (when most infestation occurs) compared to other oaks (*Q. ilex*, *Q. faginea*). This difference is probably due to its later phenology, as we have observed that when *Q. coccifera* co-exists with those two species, it flowers later. Previous studies [32] have shown this relationship between flowering phenology and acorn growth timing. *Q. pyrenaica* showed a late acorn growth phenology (acorns at T1 were proportionally the smallest compared to T2). However, this species produces the largest acorns overall, so large, in fact, that at T1, they already allowed unconstrained larval growth (cotyledon depletion by larvae was extremely rare). Large *Q. pyrenaica* acorns hence benefited insects (which could be considered a bad strategy), but this species was very effective in satiating larvae at the seed level (sensu Bonal et al. 2007 [1]). In large infested acorns, embryos (placed at the distal extreme of the acorn) are less likely to be predated and larvae leave a larger amount of cotyledon uneaten, which allows the production of larger seedlings [1]. In turn, larger seedlings are more likely to recruit, thus increasing oak fitness [57,58,59].

The combination of small size, late phenology, and high tannin content makes acorns of *Q. coccifera* a poor-quality host for pre-dispersal seed predators. However, *Q. coccifera*’s advantages against predators may turn into disadvantages when it comes to acorn dispersal. Acorn dispersal is a key stage in oak’s natural regeneration cycle and strongly influences further establishment success [60]. Birds and small mammals disperse acorns away from the mother tree, decreasing the likelihood of predation by other post-dispersal seed predators. Dispersers frequently cache acorns and, in certain cases, they do not retrieve them and acorns germinate [61,62]. Mediterranean oaks rely on dispersal, especially rodents caching acorns under shrubs, which increases seedling survival during summer drought (the main regeneration bottle-neck), and corvids, such as the Eurasian jay (*Garrulus glandarius*) and Eurasian magpie (*Pica pica)*, which are the primary dispersers of *Quercus* seeds in Mediterranean woodlands [60,63,64]. Different studies have shown that rodents select against tannin-rich acorns, which are less preferred and thus less likely to be dispersed (e.g., [65]). Moreover, smaller acorns are more likely to be eaten than dispersed [66,67]. Accordingly, a field study comparing Mediterranean oak species showed that *Q. coccifera* acorns were the least preferred acorns by dispersers, such as the Eurasian jay [68].

## 5. Conclusions

In summary, Mediterranean oak species show different strategies against insect pre-dispersal seed predators, which include satiation at the seed level, higher tannin concentrations in developing acorns, and the premature abscission of infested acorns. *Q. ilex*, *Q. faginea*, and *Q. pyrenaica* were more effective at satiating larvae at the seed level, as their acorns were larger earlier in the season (T1) when most infestation occurred. Hence, their embryos were less likely to be predated than those of *Q. coccifera*. In contrast, *Q. coccifera* was much more effective against pre-dispersal insect seed predators by constraining their larval growth. Tannin concentrations were much higher than in the rest of the *Quercus* spp., and we observed a constrained weevil larval mass, a proxy of insect fitness [52].

Moreover, its smaller acorns and late maturation phenology meant that insects frequently depleted the cotyledons in early abscised infested acorns. However, the defensive strategies against acorn-feeding insects may be counterbalanced by other selective pressures on oak fitness. For example, the costs of tannin production remain unknown in acorns, but resource allocation trade-offs in secondary chemical production have been demonstrated in other plants [69,70]. Also, a high tannin content deters acorn dispersers. All this configures a complex adaptive landscape that favors the co-existence of different strategies evolved by *Quercus* spp. against pre-dispersal insect predators.

## Figures and Tables

**Table 1:** Location and climatic characterization of the sampling sites. Annual precipitation data and average minimum and maximum temperatures were obtained using the easyclimate R package(R version 4 4 2) [35] based on 72 years of daily data in these localities.

Locality	Longitude	Latitude	Altitude	Tm	TM	aP	Species
Huecas	4°12′60″ W	39°59′60″ N	543	9.57	21.59	343.59	*Q. ilex* *Q. coccifera*
Castrejón	4°14′9″ W	39°49′49″ N	521	9.63	21.47	348.32	*Q. ilex* *Q. coccifera*
Sevilla la Nueva	4°2′57″ W	40°21′51″ N	639	9.12	20.53	376.27	*Q. ilex* *Q. faginea*
Robledillo	4°55′19″ W	40°33′11″ N	1113	5.51	16.81	380.79	*Q. ilex,* *Q. faginea* *Q. pyrenaica*
Zarzalejo	4°10′36″ W	40°33′5″ N	1146	6.67	16.62	534.67	*Q. ilex* *Q.pyrenaica*

Altitude (m.a.s.l.); Tm, mean minimum annual temperature (°C); TM, mean maximum annual temperature (°C); aP, annual precipitations (mm).

**Table 2:** The relative proportion of the total infestation (%), as well as the differences in infestation across different periods by insect taxa.

Insect Taxa	Cur	Cyd	Call	Cur + Cyd *	Cur + Call *	Cyd + Call *
Period						
All season long	31	48	12	6	2	2
T1	38	41	10	8	3	0
T2	21	58	14	3	1	4

*Curculio* spp. (Cu), *Cydia* spp. (Cyd), *Callirhytis* spp. (Call). The asterisks correspond to the proportion of acorns infested at the same time by the insect taxon indicated.

**Table 3:** The concentration of tannins, expressed as a percentage (%), occurred in all oak species and localities from T1 to T2.

		Concentration of Tannins (%)	
Locality	Species	T1	T2
Huecas	*Q. coccifera*	28.18	12.22
Huecas	*Q. ilex*	5.3	3.43
Castrejón	*Q. coccifera*	21.57	17.16
Castrejón	*Q. ilex*	6.19	5.01
Sevilla la Nueva	*Q. faginea*	4.58	4.50
Sevilla la Nueva	*Q. ilex*	2.26	1.07
Robledillo	*Q. faginea*	7.91	5.24
Robledillo	*Q. pyrenaica*	8.42	3.2
Zarzalejo	*Q. pyrenaica*	3.61	3.41
Zarzalejo	*Q. ilex*	2.24	0.98

**Table 4:** Acorn volumes (mm^3^) over maturation time for each species. Means and 95% CIs are provided for each species.

	*Q. coccifera*	*Q. faginea*	*Q. ilex*	*Q. pyrenaica*
T1	1764 ± 261	1853 ± 256	2164 ± 188	2346 ± 335
T2	3419 ± 609	2066 ± 276	2588 ± 237	6119 ± 874

## Data Availability

Data will be deposited at public repository if the article is accepted for publication.
